# Neurology

**Published:** 1891-03

**Authors:** W. C. Krauss


### NEUROLOGY.

CONDUCTED BY

W. C. KRAUSS, M. D.

In a report on the examination of one hundred brains of feeble-minded children, made by A. W. Wilmarth, of Elwyn, Pa., in the *Alienist and Neurologist* for October, 1890, the following conditions were found: Sclerosis with atrophy, 12; sclerose tuberense, 6; diffuse sclerotic change, 7; degenerative changes in vessels, ganglionic cells or medullary substance, not constituting true sclerosis, 15; hydrocephalic, 5; general cerebral atrophy, 2; non-development in various forms, 16; infantile hemorrhage, 1; extensive adhesion of membranes from old meningitis, 3; angiomatous condition of cerebral vessels (with degenerative changes), 1; glioma (with sclerosis), 1; porencephalous (with non-development), 1; of thirty-one cases where the actual disease or imperfect development of the brain proper was not demonstrated, there was hypertrophy of the skull, 6; acute softening (recent), 2; demi-microcephalic, 2; when the brain was above usual weight, but the convolutions large and very simple in their arrangement, 2.

### HYPNOTISM IN ITS RELATION TO SURGERY.

Dr. Lanphear, of Kansas City, reports a case of double talipes, in which the subject had chronic Bright's disease which contra-indicated the use of ether, and at the same time he had an organic heart trouble which presented the safe use of chloroform. The patient wanted to be operated upon, and Dr. Lanphear hesitated to give the ordinary anesthetic, and so hypnotized him. Contrary to the generally accepted idea that at the first seance a sufficient degree of anesthesia cannot be produced to perform an operation, the doctor got a sufficient degree of anesthesia by suggestion by which he performed the operation for talipes, and the patient lay upon the table as fixed and immovable as marble during the whole operation.— *Cincinnati Medical Journal.*

